# Sustainable Ultrasound-Assisted Solid-Phase peptide synthesis (SUS-SPPS): Less Waste, more efficiency

**DOI:** 10.1016/j.ultsonch.2025.107257

**Published:** 2025-02-07

**Authors:** Salvatore Mottola, Alessandra Del Bene, Vincenzo Mazzarella, Roberto Cutolo, Ida Boccino, Francesco Merlino, Sandro Cosconati, Salvatore Di Maro, Anna Messere

**Affiliations:** a Department of Environmental, Biological and Pharmaceutical Science and Technology, University of Campania “Luigi Vanvitelli”, 81100 Caserta, Italy; b Department of Pharmacy, University of Naples Federico II, 80131 Naples, Italy; c Interuniversity Research Centre on Bioactive Peptides (CIRPEB), Naples, Italy

**Keywords:** Sustainable Peptide Synthesis, Ultrasound, Sonochemistry, Solid-Phase Synthesis, Minimized solvent consumption, Green approach

## Abstract

The integration of low-frequency ultrasound with Solid-Phase Peptide Synthesis (SPPS) was explored to establish a Sustainable Ultrasound-assisted Solid-Phase Peptide Synthesis (SUS-SPPS) method. This innovative approach significantly reduces solvent consumption, washing steps, time, and reagent usage compared to conventional manual SPPS protocols. The SUS-SPPS method exploits ultrasound at every stage of synthesis and work-up, reducing the process to just two steps. The first step sequentially combines Fmoc-amino acid coupling, capping of unreacted amino groups, and Fmoc deprotection into a single operation, while the second one consists of a single washing procedure. Moreover, we demonstrated that the method is compatible with various resin types, including Rink-amide, Wang, and Cl-Trt resins, and facilitates the efficient synthesis of peptides of varying lengths (up to 20-mers) and compositions, including those traditionally considered “difficult sequences”, with excellent yields and purity. Notably, SUS-SPPS reduces solvent usage per coupling cycle by 83–88%, marking a significant breakthrough in sustainable peptide synthesis.

## Highlights

• Sustainable Ultrasound-assisted Strategy for Solid-Phase Peptide Synthesis (SUS-SPPS)
• SUS-SPPS allows to reduce the solvent consumption in peptide synthesis.
• SUS-SPPS enables the use of the most efficient solvents for the synthesis process.
• SUS-SPPS is compatible with the most commonly used resins in SPPS.
• SUS-SPPS provides excellent crude product purities and short reaction times even for difficult peptide sequences.

## Graphical abstract

**Figure:**
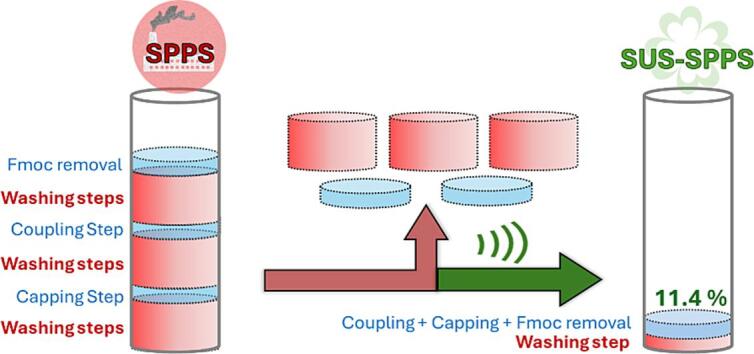

## 1 Introduction

For many years, medicinal chemists in both academia and industry have focused on developing innovative, life-enhancing drugs, placing a high priority on breakthroughs over other considerations, such as environmental impact and cost. Today, increasing awareness of the environmental effects of chemicals, along with stringent regulations from Environmental Protection Agencies (EPAs), is driving a comprehensive redesign of the entire drug discovery, development, manufacturing, and waste management process to incorporate more sustainable practices [1]. In response to this need, the close collaboration between academia and industry appears crucial in accelerating the “green transition”, with academia continuously providing new opportunities for alternative synthetic approaches, reagents, and solvents that align more closely with Green Chemistry principles. As a result, pharmaceutical companies are progressively moving from traditional [2] to efficient and sustainable synthetic methods [3], [4], [5], for the discovery and the production of the so-called Active Pharmaceutical Ingredients (APIs) [3], [4]. Peptides constitute a prominent class of active pharmaceutical ingredients (APIs), representing an increasing share of FDA-approved drugs for therapeutic, diagnostic, and preventive purposes [6], [7], [8]. As of May 2024, research in this field has resulted in the approval of more than 110 peptide-based drugs worldwide, with the market value expected to reach US$68.7 billion by 2030 [9]. Peptide drugs are used in a wide range of therapeutic areas, such as urology, respiratory therapy, pain therapy, oncology, metabolic, cardiovascular, and antimicrobial applications [10]. Their pharmacological effects are achieved through diverse mechanisms of action, such as activating or inhibiting cell surface and intracellular receptors, inhibiting intracellular enzymes, modulating protein–protein interactions, and disrupting cellular membranes. The rapid evolution of this field has been enabled by breakthroughs in structural biology, advances in recombinant technologies, and the development of innovative analytical and synthetic methods. In particular, Solid-Phase Peptide Synthesis (SPPS), introduced by Merrifield [11] and refined by subsequent researchers, has revolutionized the production of complex peptides, facilitating their large-scale manufacture. Two primary SPPS methodologies have been developed based on the strategies used to protect the α-amino group and the side-chain functional groups: the *tert*-butyloxycarbonyl/benzyl (Boc/Bzl) approach [12], [13] and the fluorenylmethoxycarbonyl/*tert*-butyl (Fmoc/tBu) approach [14]. Among these, Fmoc-SPPS has become the dominant method in modern peptide synthesis, as it facilitates milder reaction conditions for both the assembly of peptides and their cleavage from the solid support [15], [16], [17]. This has enabled the large-scale production of complex peptides like liraglutide, semaglutide, and tirzepatide for diabetes and weight management [18], [19], [20]. Despite its high potential for efficiency, SPPS still faces significant sustainability challenges due to the extensive use of solvents like *N*,*N*-dimethylformamide (DMF), *N*-methylpyrrolidone (NMP), and dichloromethane (DCM), which are hazardous and regulated under REACH guidelines [21]. A restriction on the use of DMF in the EU market, effective from December 2023, further highlights these concerns [22]. While SPPS integrates several features aligned with the 12 principles of Green Chemistry, such as minimal purification steps, no isolation of reaction intermediates, high yields, straightforward work-up and scalability, its reliance on protecting groups and substantial solvent usage remain key sustainability challenges. To reduce the use of protecting groups, different approaches have been reported including the N-acylation of unprotected α-amino acids, highly effective alternative activation of the carboxylic groups, Minimal-Protection SPPS (MP-SPPS), micro-flow solid-phase protocols, inverse peptide chemical synthesis and Nα-Smoc protecting group in aqueous SPPS (ASPPS) [23], [24], [25], [26], [27], [28], [29], [30], [31], [32], [33]. Taken together, these studies clearly indicate that resolving the issue of protecting groups often demands substantial changes to SPPS protocols and chemistry, which have been refined over decades of research. Conversely, the substantial consumption of solvents and resultant waste production could be significantly minimized by adopting green solvents compatible with the assessed SPPS protocols. In recent years, various efforts have been devoted in replacing the SPPS “gold standard” solvent DMF with greener alternatives, following established solvent-selection guidelines. Pioneering work in green solid-phase peptide synthesis (GSPPS) has been extensively documented by Ferrazzano et al. [34] and Albericio and co-workers [35], [36], [37], [38], [39], [40]. They have reported several studies focusing on the use of less hazardous solvents such as water [35], tetrahydrofuran (THF) [36], acetonitrile (ACN) [36], 2-methyltetrahydrofuran (2-MeTHF) [34], [35], cyclopentyl methyl ether (CPME) [35], ethyl acetate (EtOAc) [36], and γ-valerolactone (GVL) [39], [40]. More recently, Lopez et al. [41] reported the use of *N*-butylpyrrolidone (NBP) for synthesizing a late intermediate required for the preparation of octreotide. These advancements highlight a shift toward environmentally friendly solvents in peptide synthesis, contributing to sustainable practices in pharmaceutical research and development. However, it should be noted that an ideal solvent for SPPS must effectively facilitate resin swelling, coupling reactions, protecting group removal and washing steps. Finding a green solvent system that can optimize all these different stages simultaneously represents a hard task. As a result, many green solvents have been excluded from reported studies because they do not fulfill all these requirements adequately. To broaden the range of suitable solvents for SPPS, the use of solvent mixtures has been explored [42], [43], highlighting the difficulty of green solvent selection in SPPS and the need of proper solvent systems that can be suitable for each step of the synthetic process. On the other hand, “The best solvent is sometimes no solvent”, which perfectly aligns with the fifth principle of Green Chemistry [2] that advocates for elimination of solvents whenever feasible. It has been shown that many chemical syntheses can be conducted without the need for solvents by applying the mechanochemistry approach [44], [45], [46], [47]. Although eliminating solvents may not be always achievable, significantly reducing their usage, particularly by selecting the most effective solvent for each task, can be a profoundly impactful and environmentally friendly approach. On the other hand, many pieces of evidence support the application of green technologies that enhance SPPS chemical transformations using sustainable energies, thereby reducing solvents, reagents, costs, and reaction times [47]. Among them, microwave-assisted methodology fits within Green Chemistry principles due to the efficient energy use and shorter reaction times provided by microwave irradiation. The use of flow systems has also enabled the scale-up of these microwave-assisted reactions in an eco-friendly manner [48]. Microwave peptide synthesizers enable faster reactions and improved yields but face challenges such as high costs, degradation of sensitive reagents at elevated temperatures, solvent incompatibilities, and limitations with certain peptide modifications. These issues may reduce efficiency, yield, and purity, requiring careful consideration before adoption [48], [49]. Alongside the early reports of energy-assisted reactions, numerous studies have explored the use of ultrasounds (US) to activate reagents in both homogeneous and heterogeneous reactions. This research has led to the emergence of a branch of chemistry known as “sonochemistry” [50], [51]. Ultrasonication is a highly regarded eco-environmental technology, based on the phenomenon of cavitation, which generates extremely high local temperatures (around 5000 K) and pressures (over 1000 atmospheres) in a liquid phase. In organic synthesis, this method offers several advantages over heating procedures: increased reaction kinetics, improved product quality, yields, and selectivity (known as “sonochemical switching”), reduced reaction times, limited energy consumption and waste production, the ability to perform reactions with non-classical solvents (e.g., PEG and water instead of volatile organic solvents) or without solvents, milder conditions for both homogeneous and heterogeneous reactions [52], [53], [54], [55], [56], [57], [58], [59], [60], [61], [62]. The synergy between low-frequency ultrasounds and solid-phase peptide synthesis was previously demonstrated, leading to development of the US-SPPS method, which significantly reduced material usage and reaction times, greatly improving the efficiency of individual SPPS reactions [56], [57], [58], [62]. Inspired by these studies, we aimed to develop an easily accessible, efficient, and sustainable method for ultrasound-assisted SPPS. Herein, by systematically applying ultrasonication across all stages of SPPS, including washing steps, we were able to significantly reduce the solvent consumption, the number of washing cycles, the overall synthesis time, and reagent usage compared to conventional SPPS methods. This effort culminated in the creation of a more sustainable approach, which we named Sustainable UltraSound-assisted SPPS (SUS-SPPS).

## 2 Results and discussion

**Fig. 1:**
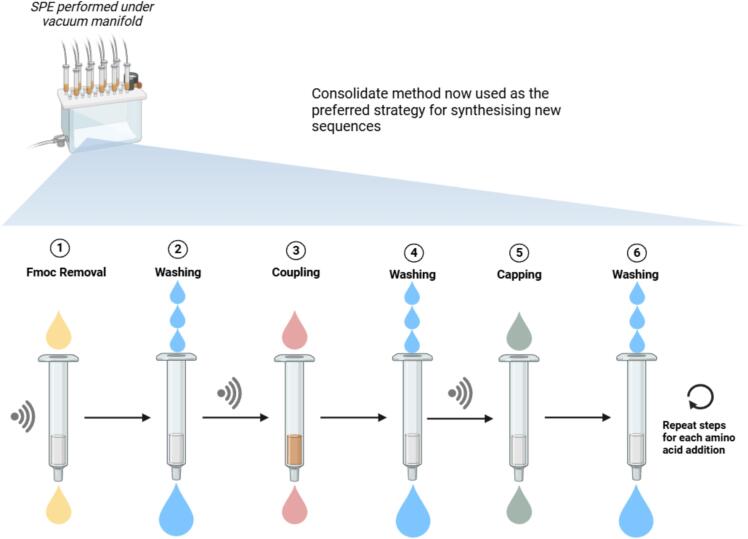
Schematic overview of the conventional SPPS process.

Introducing a single amino acid in Fmoc-SPPS can be a cumbersome operation, which requires the following phases (Fig. 1): (1) Fmoc removal through a double treatment with 20 % piperidine in DMF; (2) three to five washings with DMF to remove the excess of piperidine and *N*-fluorenylmethylpiperidine; (3) coupling of Fmoc-aa-OH with a proper activation strategy; (4) three to five washings with DMF to eliminate the excess of reagents and by-products; (5) optional capping step using a solution 1:1:3 of acetic anhydride, DMF and pyridine or DIPEA; (6) further three washings with DMF to remove the excess of acetic anhydride, the resulting acetic acid and the base [63]. Consequently, to ensure the efficient removal of excess reagents and potential side products from the reaction vessel, a minimum of ten washing steps is required. This substantially increases the total waste generated and contributes to a high process mass intensity (PMI) [64], [65].

**Fig. 2:**
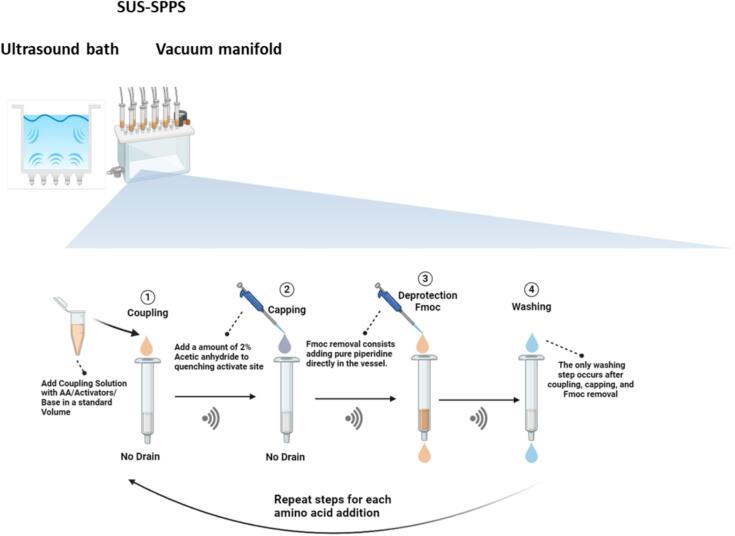
Schematic overview of the SUS-SPPS process.

Delving deeper into the SPPS process, it is important to highlight that, beyond the steps of Fmoc removal and amino acid activation, required for deprotecting the peptidyl-bound resin and acylating the resulting α-NH_2_ group, respectively, the washing operations in step (2) play a critical role. These washes are essential for removing piperidine excess, which could otherwise impair the SPPS performance by prematurely detaching the Fmoc group from the subsequent Fmoc-aa-OH. Similarly, although the capping step is often considered optional throughout SPPS, acetylating the N-terminal region is essential for peptides intended to mimic a specific protein or peptide segment. Additionally, acetylation is particularly beneficial for reducing deletion products when synthesizing difficult or long peptides, significantly enhancing the quality of the crude peptide and streamlining the purification process. Thus, considering that in 2016 the ACS Green Chemistry Institute Pharmaceutical Roundtable indicated reducing the environmental impact of SPPS as a critical unmet need, efforts to reduce solvent consumption have primarily focused on eliminating all solvent-intensive washing steps during each amino acid addition cycle [66], [67]. On this basis, inspired by one of the synthetic programs developed by CEM [68], Albericio’s team combined two steps into one by applying the *in situ* Fmoc removal strategy [69]. After the coupling process, they added 20 % piperidine or 4-methylpiperidine within the coupling mixture. Further, they demonstrated that washing with a weak acid (1 % OxymaPure) effectively removed piperidine traces. This approach reduced solvent use by 75 %. More recently, the *in-situ* Fmoc removal protocol was optimized by incorporating an extra base treatment with 4-methylpiperidine, to ensure complete Fmoc removal, requiring additional carbodiimide during the coupling step to boost active ester formation by *in situ* activation [70]. Meantime, the research team at CEM Corporation introduced an SPPS method using microwave energy and heating [71] eliminating all solvent-intensive washing steps during each amino acid addition cycle. A key innovation is the removal of the volatile Fmoc deprotection base via bulk evaporation at high temperatures, combined with directed headspace gas flushing to prevent condensation on vessel surfaces. In line with these studies, to explore the potential of ultrasonication within an environmental framework for reducing solvent consumption and enhancing the sustainability of SPPS, we redesign the entire SPPS coupling/deprotection cycle by combining the coupling, capping, and Fmoc removal steps in sequence and by performing a single wash at the end of the procedure (Fig. 2). Additionally, COMU/OxymaPure combination was selected as a coupling strategy because it is widely regarded as the optimal choice for manual SPPS, offering an excellent balance of efficiency, cost-effectiveness, and reduced environmental impact [72]. Upon completion of sequence elongation, deprotection and cleavage steps were carried out by trifluoroacetic acid (TFA) − triisopropylsilane (TIS)–H_2_O (95:2.5:2.5). The residue was precipitated with cold ether to obtain the crude peptide, which was later analyzed by RP-HPLC and LC-MS. The performance of the strategies was assessed by comparing the HPLC profile of the crude products obtained by the same protocols with or without ultrasonication.

### 2.1 SUS-SPPS of model pentapeptide Fmoc-KFRFD-NH_2_

We conducted a preliminary screening of various synthetic procedures, which differed in reaction and washing volumes as outlined in Table 1, by synthesizing a model pentapeptide (Fmoc-KFRFD-NH_2_) using Rink amide-AM PS resin (0.64 mmol/g). The pentapeptide was assembled on a 0.05 mmol scale (78.12 mg of resin) in the presence of 2 equiv of Fmoc-aa-OH/COMU/Oxyma and 4 equiv of DIPEA.

**Table 1:** Reaction conditions for the synthesis of Fmoc-KFRFD-NH_2_ model peptide. N.A. = Not Applied.

		**Method**				
	***a***	***b***	***c***	***d***	***e***	***f***
**Coupling**	DMF 400 μL, 15 min	DMF 800 μL, 15 min	DMF 1200 μL, 15 min	DMF 400 μL, 15 min	DMF 800 μL, 15 min	DMF 1200 μL, 15 min
**Capping**	Ac_2_O 2 %, 3 min	Ac_2_O 2 %, 3 min	Ac_2_O 2 %, 3 min	Ac_2_O 2 %, 3 min	Ac_2_O 2 %, 3 min	Ac_2_O 2 %, 3 min
**Deprotection**	20 %Pip in DMF 100 μL, 2 min	20 %Pip in DMF 200 μL, 2 min	20 %Pip in DMF 300 μL, 2 min	20 %Pip in DMF 100 μL, 2 min	20 %Pip in DMF 200 μL, 2 min	20 %Pip in DMF 300 μL, 2 min
**Washing**	DMF 1 x 400 μL, 5 min	DMF 1 x 800 μL, 10 min	DMF 1200 μL, 10 min	DMF 1 x 400 μL, 5 min	DMF 800 μL, 10 min	DMF 1200 μL, 10 min
**Ultrasonication**	YES	YES	YES	N.A.	N.A.	N.A.

**Fig. 3:**
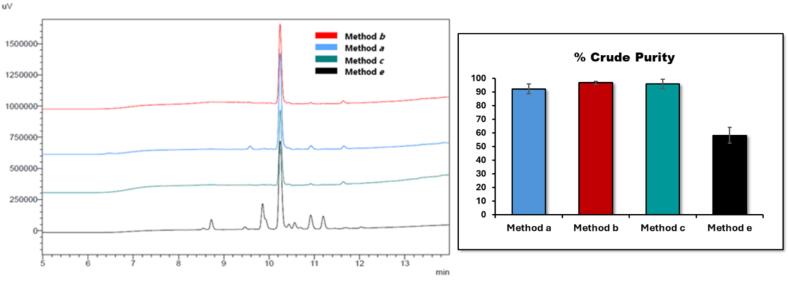
Optimization study for the SUS-SPPS of the model 5-mer peptide (Fmoc-KFRFD-NH_2_). Peptide synthesis was attained in triplicate and the resulting crude purities are expressed as a percentage (mean values ± standard error of measurement (SEM), N = 3).

Briefly, Fmoc-aa-OH, COMU-OxymaPure, and DIPEA were dissolved in DMF (400 μL for method ***a***, 800 μL for method ***b***, and 1200 μL for method ***c***) and added to the amino functionalized solid support. After 15 min of US-assisted coupling, pure acetic anhydride (2 %) was added with respect to the volume of DMF and DIPEA already present in the reaction vessel. After an additional 3 min under US irradiation, pure piperidine (100 μL for method ***a***, 200 μL for method ***b***, and 300 μL for method ***c***) was added to the coupling mixture and allowed to react for 2 min under US irradiation. Finally, the peptide-functionalized support was washed once with DMF (400 μL for method ***a***, 800 μL for method ***b***, and 1200 μL for method ***c***) with a 5 min ultrasonic treatment. Comparison of the resulting HPLC profiles of crude pentapeptides (Fig. 3) indicated that method ***b*** (and ***c***) was the most effective. Noteworthy, the comparison between the purity of the crude products obtained by methods ***b*** with those achieved through mechanical shaking (Fig. 3, methods ***e***) clearly pointed out the beneficial effects of ultrasonication not solely on the coupling and Fmoc removal processes but also on the washing operation.

**Fig. 4:**
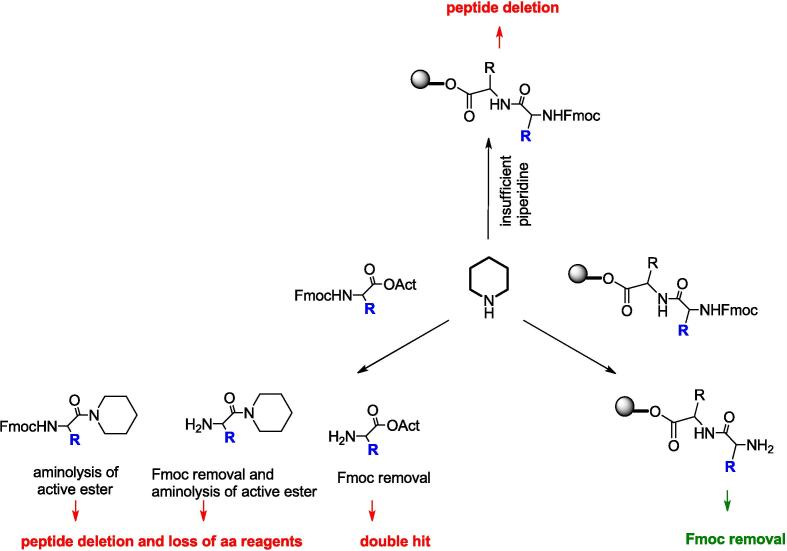
Unwanted and Desired Effects of Adding Piperidine at the End of the Coupling Step.

Most importantly, our results suggested that ultrasonication can play a significant role in suppressing two potential piperidine-based competitive reactions, namely the peptide deletion and the double hit (Fig. 4). Indeed, when added subsequently to Fmoc-aa/coupling agents mixture in the presence of ultrasound, piperidine promotes the removal of Fmoc from the peptidyl-resin and deactivates the remaining amino acid excess through aminolysis, reducing the risk of partial or incomplete Fmoc removal, the formation of truncated sequences or the introduction of undesired residues in the sequence (Fig. 4).

It is plausible to assume that ultrasound primarily accelerated deactivation of the active ester more easily than the removal of Fmoc, thus preventing the formation of peptides with multiple hit due to the over-acylation of amino acids and free amino peptidyl resin. On the other hand, the effectiveness of Fmoc removal from the peptidyl-bound resin by ultrasonication prevents the formation of both Fmoc-protected and deleted peptides. More importantly, ultrasonication also enables effective washing of the resin by promoting gentle yet effective stirring, which facilitates the removal of byproducts and piperidine traces from the resin. Interestingly, no undesired reactions were observed when pure acetic anhydride and piperidine were sequentially added to the same reaction mixture. This was confirmed by comparing the HPLC profiles of the crude Fmoc-KFRFD-NH_2_ obtained with and without the capping step (see Fig. S4 in S.I.). Reasonably, adding piperidine was sufficient to both neutralize acetic anhydride and promote Fmoc-removal in the short times of ultrasound-assisted reactions, eliminating the need for quenching treatments beyond simple washing. Finally, it is worth noting that the ultrasound-assisted coupling did not require further addition of activating agents during the reaction to compensate for the possible loss of active ester.

Given the high and comparable purity of the crude products (≥ 96 % both ***b*** and ***c*** methods) and considering required lower volume of DMF, we chose method ***b*** to synthesize other biologically relevant peptides (Table 2).

**Table 2:** Synthesized Biologically Relevant Sequences by US-SPPS method.

**Entry**	**Peptide**	**US assisted-method**	**t_r_ min**	**Crude purity SUS-SPPS (%)**	**Not US-assisted method**	**Crude purity SPPS (%)**
**1**	Fmoc-KFRFD-NH_2_	b	10.2^a^	97 ± 3	e	47 ± 2
**2**	YNWNSFGLRF (Kisspeptin_10_)	b	9.5	95 ± 3	e	11 ± 3
**3**	ARLDVASEFRKKWNKWALSR (PAMP_1-20_)	c	15.0	83 ± 4	f	34 ± 3
**4**	YAib-Aib-FL (Aib-Enkephaline)	c	16.4	88 ± 1	f	17 ± 3
**5**	VQAAIDYING (ACP_65−74_)	c	13.6	98 ± 3	f	71 ± 4
**6**	DRVYIHPFHL-COOH (Angiotensin-I)	b	15.2	93 ± 3	e	14 ± 3
**7**	LRKYRP-COOH	b	9.8	90 ± 2	e	57 ± 4

### 2.2 SUS-SPPS of biological relevant peptides

To validate the feasibility of the SUS-SPPS method, we tested it for the synthesis of some biological relevant peptides by comparing the crude purity percentage of the peptides obtained by SUS-SPPS and conventional SPPS methods (Table 2).

The optimized SUS-SPPS protocols for peptide sequences **1**–**7** were detailed in Table 1. Peptides were synthesized as follow: entries **1** (methods *a*, *b*, *c*, *e*, Table 1) and **2** (methods *b* and *e*, Table 1) on Rink amide resin 0.64 mmol/g; 78 mg, 0.05 mmol; entries **3**–**5** (methods *c* and *f*, Table 1) on Rink amide resin LL 0.29 mmol/g; 172 mg, 0.05 mmol; entry **6** (method *b* and *e*, Table 1) on Wang resin 0.64 mmol/g pre-loaded with Fmoc-Leu amino acid; 39 mg, 0.025 mmol, entry **7** (methods *b* and *e*, Table 1) on 2-CTC resin 0.50 mmol/g pre-loaded with Fmoc-Leu amino acid; 100 mg, 0.05 mmol.

**Fig. 5:**
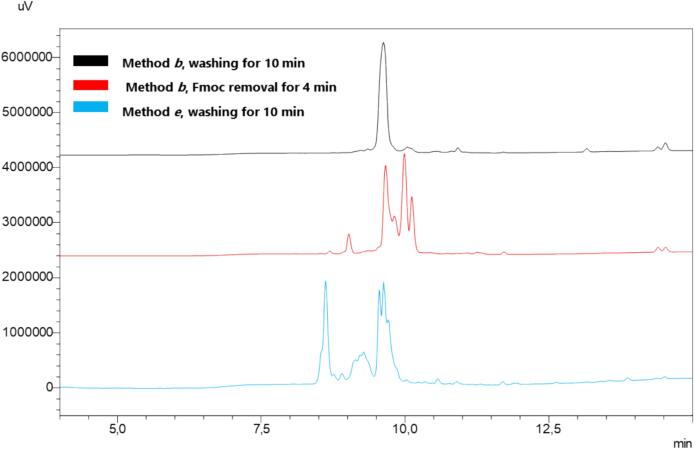
SUS-SPPS of peptide Kisspeptin10 (YNWNSFGLRF). Peptide synthesis was attained in triplicate and the resulting crude purities are expressed as a percentage (mean values ± standard error of measurement (SEM), N = 3).

Kisspeptin10 (YNWNSFGLRF) (entry **2,** Table 2) [73] with amide C-terminal, was assembled by applying method ***b*** (US-assisted) and compared with method ***e*** (not US-assisted). As depicted in Fig. 5, The HPLC profiles of the crude Kisspeptin10 showed the presence of the desired peptide, as confirmed by LC-MS, but unfortunately, with an unsatisfactory degree of purity as indicated by the presence of several side products. Assuming that the lack of efficiency could be correlated with the capability of piperidine, to remove Fmoc protecting group from longer peptidyl bound resin, we initially attempted at increasing the Fmoc removal time in the method ***b***, from 2 to 4 min but Kisspeptin_10_ peptide did not result as the main product of the crude (Fig. 5). At this stage, we decided to modify the washing time after Fmoc removal, extending it from 5 to 10 min in the method ***b***. As a result, without increasing the volume of solvent or the number of washes, we improved the purity (>95 %) of the crude of Kisspeptin_-10_, as shown by the HPLC profile (Fig. 5, method ***b***, 10 min washing).

**Fig. 6:**
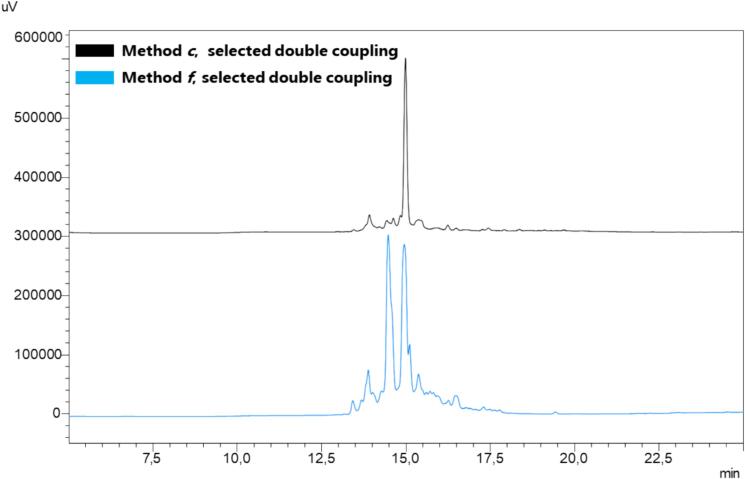
SUS-SPPS of peptide PAMP_1-20_ (ARLDVASEFRKKWNKWALSR). Peptide synthesis was attained in triplicate and the resulting crude purities are expressed as a percentage (mean values ± standard error of measurement (SEM), N = 3).

Along the same line of intervention, we adopted the same scale for the synthesis of PAMP 1–20 (entry **3**, Table 2) [74], (Rink amide-AM PS resin, 0.29 mmol/g, 156,24 mg, 0.05 mmol). We compared the ultrasound method to mechanical shaking under identical conditions of reagents, volumes, and times. To ensure adequate suspension of the larger quantity of resin, we applied method ***c***, which involved coupling in 1200 μL DMF, capping by addition of 2 % of acetic anhydride after coupling, then addition of 300 μL piperidine for Fmoc removal, and finally, a single wash whit 1200 μL of DMF for 10 min. Additionally, the synthesis of PAMP_1-20_ was accomplished by employing selective double coupling for Ala, Arg, and the amino acids following the aromatic residues. The HPLC profiles (Fig. 6) showed the desired peptide with excellent purity (approximately > 83 %).

The limitations encountered during the synthesis of long peptides (≥ 20 mer) appeared even more evident for peptides endowed with “difficult sequences”, whose synthesis is affected by three common issues, including incomplete couplings, difficulties in the loading of sterically hindered amino acids, and partial Fmoc removal [75], [76], [77]. Thus, the SUS-SPPS method was tested on two well-known “difficult sequences”, including Enkephaline, Aib-Enk, (YAib-Aib-FL, entry **4**, Table 2) [78] and the Acyl Carrier Protein fragment ACP_65−74_ (VQAAIDYING, entry **5**, Table 2) [79]. In particular, the Aib-Enk peptide is often selected as model to test the efficiency of new synthetic methos because the incorrect inclusion of Aib residues can easily occur during synthesis. Likewise, ACP_65−74_ is frequently used as a model due to its propensity for aggregation, Val deletion and folding during SPPS, which can lead to synthesis challenges [79], [80].

**Fig. 7:**
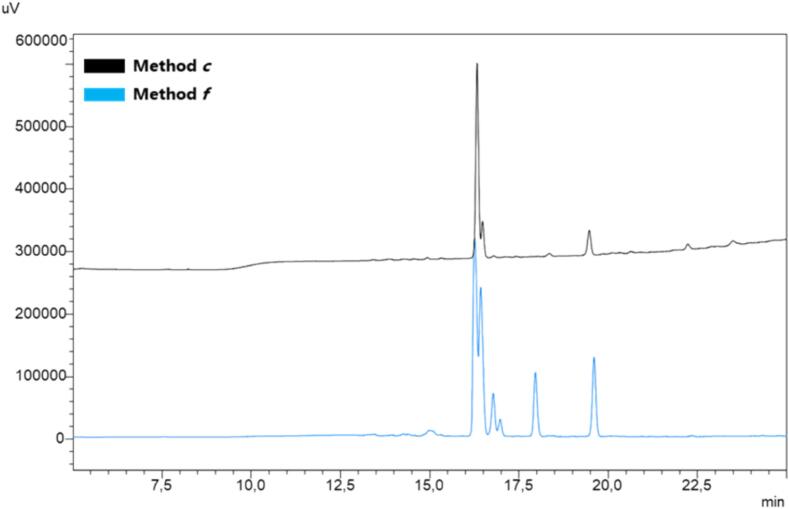
SUS-SPPS of peptide Aib-Enk, (YAib-Aib-FL). Peptide synthesis was attained in triplicate and the resulting crude purities are expressed as a percentage (mean values ± standard error of measurement (SEM), N = 3).

The synthesis of Aib-Enk was performed using method ***c*** with the scale of reaction adopted for PAMP 1–20 (Rink amide-AM PS resin, 0.29 mmol/g, 156,24 mg, 0.05 mmol) but employing 4 equiv of Fmoc-AA-OH/COMU/Oxyma and 8 equiv of DIPEA. Once again, HPLC analysis confirmed the robustness of our method, revealing a chromatographic profile with a reduced number of peaks associated with by-products compared to conventional approach, even for this challenging sequence (Fig. 7).

**Fig. 8:**
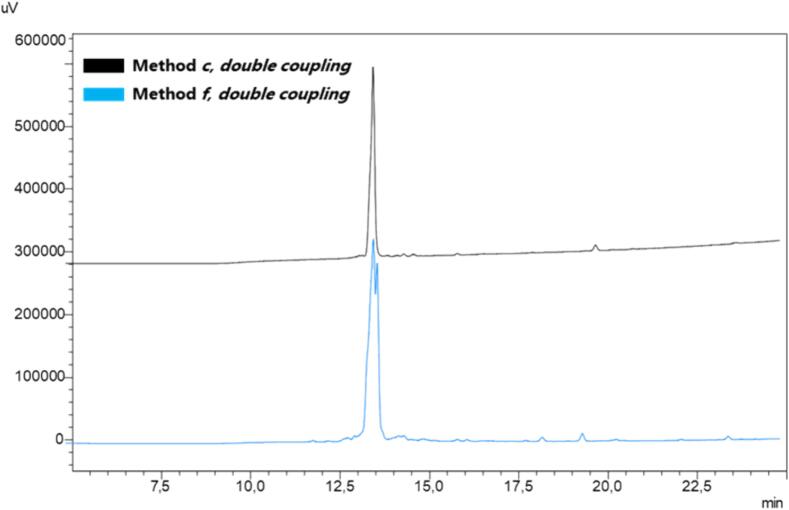
SUS-SPPS of peptide ACP_65−74_ (VQAAIDYING). Peptide synthesis was attained in triplicate and the resulting crude purities are expressed as a percentage (mean values ± standard error of measurement (SEM), N = 3).

The ACP_65−74_ peptide was synthesized applying method ***c*** and double coupling, as commonly required for this difficult sequence [76], [77], [78], [79], [80] (Fig. 8). The HPLC profiles of the resulting peptides showed the desired peptides with excellent purity (approximately 88 % for Aib-Enk and 98 % for ACP_65−74_, respectively), providing strong evidence of the substantial benefits of SUS-SPPS, particularly for aggregation-prone sequences.

**Fig. 9:**
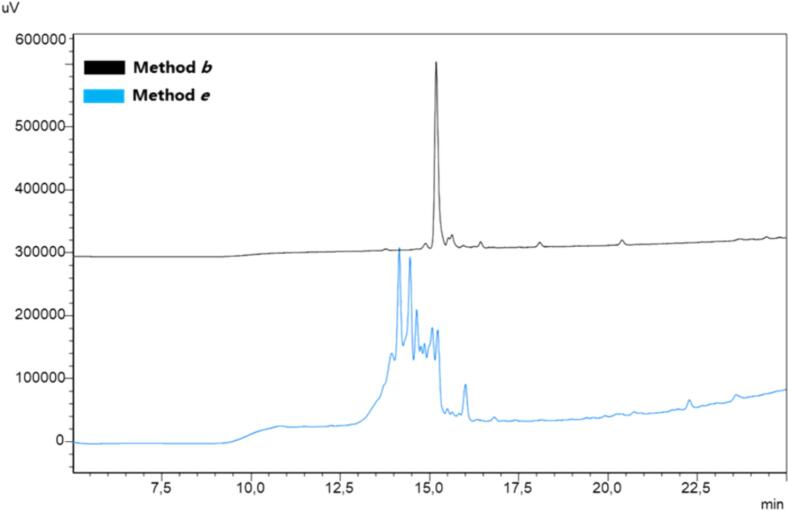
SUS-SPPS of peptide Angiotensin-I (DRVYIHPFHL-COOH) on Wang resin. Peptide synthesis was attained in triplicate and the resulting crude purities are expressed as a percentage (mean values ± standard error of measurement (SEM), N = 3).

**Fig. 10:**
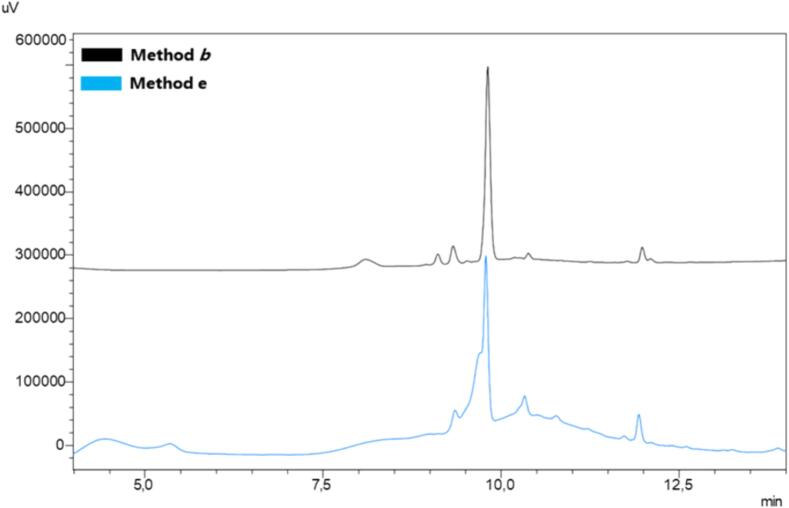
SUS-SPPS of peptide Leu-Arg-Lys-Tyr-Arg-Phe on TRT-Cl resin. Peptide synthesis was attained in triplicate and the resulting crude purities are expressed as a percentage (mean values ± standard error of measurement (SEM), N = 3).

To proceed with the investigation and to explore the flexibility of our method to different resin bound linkers, we expanded its application to the synthesis of CO_2_H-terminal peptides. Thus, we adopted the method ***b***, to synthesize Angiotensin-I [81] (DRVYIHPFHL-CO_2_H, entry **6**, Table 2) and LRKYRP-CO_2_H hexamer (entry **7**, Table 2 by using pre-loaded Wang (0.64 mmol/g) and 2-CTC resins (0.50 mmol/g), respectively. As shown in the HPLC profiles, the crude peptides (Fig. 9, Fig. 10) were obtained in high purity (92 % and 90 %, respectively), free of significant byproducts such as aspartimide and epimers, as previously demonstrated for ultrasound-supported SPPS [56], [62].

Taken together, our results prove the reliability of the SUS-SPPS strategy, based on low-frequency ultrasound, which allows for reduced solvent consumption over the full synthetic process, regardless of the type of linkers bonded to the resin.

We demonstrated that the assistance of ultrasounds during each step of the synthesis is not only compatible with Rink-amide, Wang, and 2-CTC resins, but also significantly reduces reaction volumes and requires just a single washing step after coupling, capping, and Fmoc removal. This approach enabled the efficient synthesis of peptide molecules up to 20-mer, including challenging sequences, with high yields and excellent purity. The US-SPPS strategy results in a solvent saving of 83–88 %, each coupling cycle.

### 2.3 Scale-up, sustainability, and cost-effectiveness evaluation of the SUS-SPPS method

**Fig. 11:**
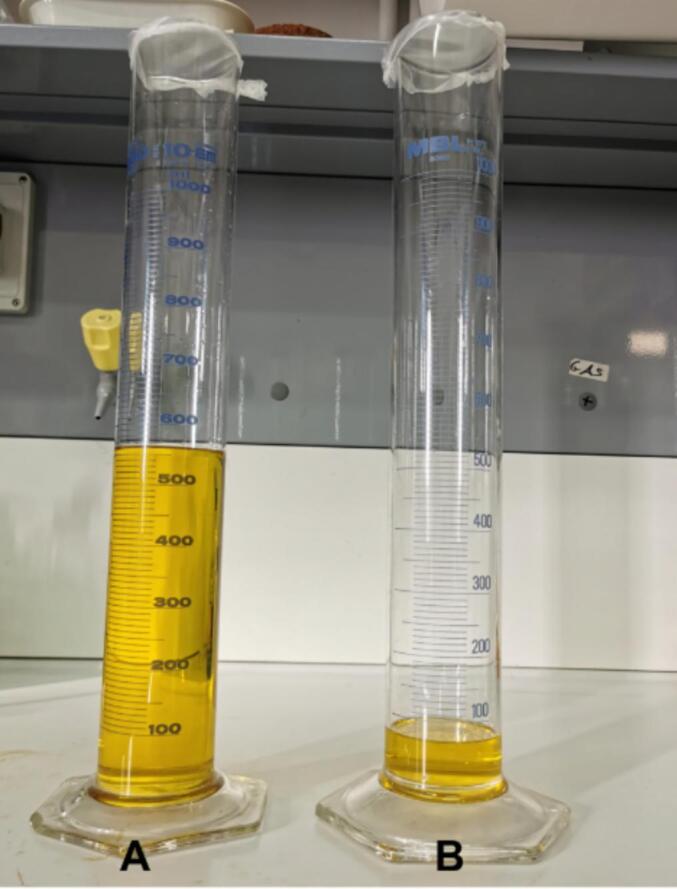
Solvent waste during the synthesis of Fmoc-KFRFD-NH_2_ on 0.5 mmol scale. **A**: standard protocol, **B**: SUS-SPPS protocol ***b*** (Table 2).

One of the critical issues in sustainable peptide-based drug development is scaling up the synthetic strategy from the laboratory to a commercially feasible level. In this section, we focused on quantifying the green credentials of the SUS-SPPS method. For this purpose, we successfully synthesized Fmoc-KFRFD-NH_2_ on a sub-mmolar scale (0.5 mmol, 0,78 g) using the SUS-SPPS method and applying protocol ***b***. After preparative HPLC purification, the resulting pure peptide was obtained in 52 % yield. We also compared the DMF consumption of the SUS-SPPS method with that of the standard manual synthesis protocol. The SUS-SPPS approach generated only 64 mL of waste, which is one tenth of the volume required for conventional synthesis (562 mL) (Fig. 11). This substantial reduction in waste demonstrates that the SUS-SPPS method is a greener alternative for SPPS. Based on DMF prices in September 2024 [82] (North America: US$ 1.38/kg, Europe: US$ 1.46/kg, Northeast Asia: US$ 0.57/kg, and India: US$ 1.91/kg, with an average of US$ 1.33/kg), we calculated a saving of 88.6 % (equivalent to an expense of 0.15 US$/Kg). Key metrics for assessing the environmental impact of a synthetic process include the process mass intensity (PMI = mass of reactants/mass of product) and the environmental factor (E-Factor = mass of waste/mass of product) [83], [84]. These metrics were calculated for the 0.5 mmol scale synthesis of Fmoc-KFRFD-NH_2_ using the SUS-SPPS method and compared to those of standard SPPS (which uses five washes between each step and 3 equivalents of the coupling cocktail). The PMI and E-Factor for SUS-SPPS were 385.29 and 369.56, respectively, while for standard SPPS they were 2915.90 and 2900.16, respectively. This notable reduction (around 7.5 times) in PMI and E-Factor highlights the sustainability of the SUS-SPPS protocol for efficient peptide synthesis without compromising yield or purity.

## 3 Conclusions and outlook

In conclusion, the SUS-SPPS method presents a more sustainable and eco-friendlier alternative to conventional solid-phase peptide synthesis, providing a cost-effective solution even for large-scale peptide production. This ultrasound-based technique not only reduces solvent and reagent costs but also saves time, by preserving the use of the most effective solvent for these types of synthetic reactions. The improvements achieved underscore the potential of SUS-SPPS to meet the rigorous sustainability demands of modern drug development and manufacturing. We anticipate that further refinements to this protocol will enhance the green chemistry toolkit for SPPS, making the approach even more sustainable and adaptable to other peptide-based biomolecules, such as peptide nucleic acids (PNA).

Author contributions

All authors have given approval to the final version of the manuscript.

## CRediT authorship contribution statement

**Salvatore Mottola:** Writing – review & editing, Writing – original draft, Supervision, Project administration, Investigation, Funding acquisition, Data curation, Conceptualization. **Alessandra Del Bene:** Methodology, Investigation, Data curation. **Vincenzo Mazzarella:** Methodology, Investigation. **Roberto Cutolo:** Methodology, Investigation. **Ida Boccino:** Methodology, Investigation. **Francesco Merlino:** Writing – review & editing, Methodology, Investigation, Funding acquisition. **Sandro Cosconati:** Writing – review & editing, Supervision, Conceptualization. **Salvatore Di Maro:** Writing – review & editing, Writing – original draft, Supervision, Project administration, Investigation, Funding acquisition, Data curation, Conceptualization. **Anna Messere:** Writing – review & editing, Writing – original draft, Supervision, Project administration, Methodology, Investigation, Funding acquisition, Data curation, Conceptualization.

## Declaration of competing interest

The authors declare that they have no known competing financial interests or personal relationships that could have appeared to influence the work reported in this paper.
