# Supplementary material for: Sustainable Ultrasound-Assisted Solid-Phase peptide synthesis (SUS-SPPS): Less Waste, more efficiency
Source: Ultrason Sonochem. 2025 Feb 7;114:107257. doi: 10.1016/j.ultsonch.2025.107257 (PMC11849595; doi:10.1016/j.ultsonch.2025.107257)
Supplement: Supplementary Data 1 [file mmc1.docx]

**Sustainable Ultrasound-Assisted Solid-Phase Peptide Synthesis (SUS-SPPS): Less Waste, More Efficiency**

Salvatore Mottola^1^, Alessandra Del Bene^1^, Vincenzo Mazzarella^1^, Roberto Cutolo^1^, Ida Boccino^1^, Francesco Merlino^2^, Sandro Cosconati^1^, Salvatore Di Maro^*,1,3^ and Anna Messere^*,1,3^

e-mail: [salvatore.dimaro@unicampania.it](mailto:salvatore.dimaro@unicampania.it); [anna.messere@unicampania.it](mailto:anna.messere@unicampania.it)

^1^Department of Environmental, Biological and Pharmaceutical Science and Technology, University of Campania “Luigi Vanvitelli”, 81100 Caserta, Italy;

^2^Department of Pharmacy, University of Naples “Federico II”, 80131, Naples, Italy.

^3^Interuniversity Research Centre on Bioactive Peptides (CIRPEB), Naples, Italy

**Table of Contents**

1. Experimental Procedures S2

1.1 Materials and general procedures S2

2. US-SPPS: Protocol and procedures S3

2.1 Optimization and SUS-SPPS of peptide sequences 1-7 S4

1. HPLC Chromatograms and Mass Spectra S5

**1. Experimental Procedures**

**1.1 Materials and general procedures**

The ultrasonic bath was SONOREX RK 52 H (inner dimensions 150×140×100 mm and operating volume 1.2 L) by Bandelin electronic (Germany), equipped with timer control for 1-15 minutes and continuous (∞) operations and built-in thermostatically adjustable heating control (30-80 °C) . Ultrasonic frequency was 35 kHz. Ultrasonic frequency is 35 kHz. The ultrasonic nominal output was 60 W. The ultrasonic peak output was 240 W, equivalent to 4 times ultrasonic nominal output. The heating power was 140 W. All standard N-Fmoc α-amino acids were from Sigma-Aldrich-Merck, (Darmstadt, Germania). The unconventional amino acid Fmoc-Aib-OH was purchased from Iris-Biotech GmbH (Marktredwitz, Germany). Piperidine (peptide grade, purity 99.9%), Trifluoroacetic acid (TFA) (peptide grade, purity 99.9%), Triisopropylsilane (TIS) (purity >97%) and N,N-diisopropylethylamine (DIPEA) (purity 99.7%) were purchased from Sigma-Aldrich-Merck, (Darmstadt, Germania). Coupling reagents such as 1-cyano-2-ethoxy-2-oxoethylidenaminooxy)dimethylaminomorpholino-carbenium hexafluorophosphate (COMU) (purity 97%), ethyl cyano(hydroxyimino)acetate (Oxyma) (purity 97%), were purchased by Sigma-Aldrich-Merck, (Darmstadt, Germania). Acetic anhydride (Ac_2_O, purity >98%) was purchased from Sigma-Aldrich (Milano, Italy). The resins Fmoc Rink amide (aminomethyl)polystyrene (AM-PS) (0.64 mmol/g as loading, 100- 200 mesh as particle size), LL Fmoc Rink amide (aminomethyl)polystyrene (AM-PS) (0.29 mmol/g as loading, 100-200 mesh as particle size) and Fmoc-L-Leu-Wang (0.64 mmol/g as loading, 100-200 mesh as particle size) were purchased by Iris-Biotech GmbH (Marktredwitz, Germany). The Fmoc-L-Leu- 2-chlorotrityl chloride resin (2-CTC, 0.50 mmol/g as loading, 100-200 mesh as particle size) was purchased by Abcr (Karlsruhe, Germany) and the manufacturer’s reported loading of the resin was used in the calculations. Solvents for peptide synthesis and analysis such as N,N-Dimethylformamide (DMF), Dichloromethane (DCM), Diethyl ether (Et_2_O), Water and Acetonitrile (MeCN) for HPLC were acquired from commercial sources (Sigma-Aldrich/Merck, Milano, Italy). All solvents were not anhydrous and used without further purification. Analytical HPLC analyses were performed by reverse-phase HPLC (Shimadzu Model SPD-40V) on a Phenomenex Kinetex column (C18, 150 mm × 4.6 mm, 5 μm, 100 Å) with a flow rate of 1 mL/min, with detection at 220 and 254 nm wavelengths by a UV-Vis detector, and by using different gradient elutions of MeCN (0.1% TFA) in water (0.1% TFA). Mass measurements were acquired by LC/MS system (LCMS-2020, Shimadzu) at the flow rate of 0.2 mL/min, and proton adducts, [M+H]^+^ , were used for empirical formula confirmation.

**HPLC and HPLC-MS methods**

1. Gradient 1：

Flow A: 0.1% TFA in H_2_O; Flow B: 0.1% TFA in MeCN.

Ratio: 10-90% B in 10 min.

Flow rate: 1 mL/min

Column: Phenomenex® C18 5 × 150 mm column

Record wave length: 220 nm

1. Gradient 2：

Buffer A: 0.1% TFA in H_2_O; Buffer B: 0.1% TFA in MeCN.

Ratio: 10-90% B in 20 min.

Flow rate: 1 mL/min

Column: Phenomenex® C18 5 × 150 mm column

Record wave length: 220 nm

**2. US-SPPS: Protocol and procedures**

Peptides were assembled using the laboratory equipment shown in Figure S1. In particular, each peptide sequence was synthesized on suitable solid support placed in a 10 mL polypropylene tube (Figure S1a) (ISOLUTE® SPE filtration column by Biotage, Uppsala, Sweden) equipped with filter (ISOLUTE® frits, 20 μm porosity polyethylene frits by Biotage, Uppsala, Sweden), stopper, and top cap. The resin was swollen for 30 min at RT on a magnetic stirrer plate (Figure S1b) (MR Hei-Standard by Heidolph, Schwabach, Germany). Then, a solid-phase extraction (SPE) vacuum manifold (Figure S1c) (Phenomenex, Torrance, USA) was used for filtering and washing procedures of the resin by means of a PTFE universal stopcock that allowed the solvent passing through the resin and collected in a waste container. The synthetic method consisted of cycles of peptide bond formation (coupling), in situ capping and Nα-Fmoc removal reactions performed in an ultrasonic bath (Figure S1d) (SONOREX RK 52 H by BANDELIN electronic, Germany). Ultrasonic irradiation during SPPS reactions was performed by placing the polypropylene vessel containing the reaction mixture whose level did not exceed that of the water in the ultrasonic bath. The filtering and washing procedures were carried out by the SPE vacuum manifold. For the pre-loaded resin, Fmoc removal was achieved by applying the Fmoc deprotection (method *b)*. Coupling was carried out in DMF (0.8 ml for method *b*; 1.2 mL for method *c*) and 2 eq. of Fmoc-amino acid, 2 eq. of activing reagents (COMU/Oxyma) and 4 eq. of DIPEA, under ultrasound irradiation for 15 minutes. Next, acetic anhydride 2% v/v was added in the reactor containing coupling solution (16 uL for method *b*; 24 uL for method *c*) and kept for 2 min under US irradiation. Then, Fmoc removal was accomplished by adding pure piperidine in the vessel (200 uL for method *b;* 300uL for method *c*) in 2 min under ultrasound irradiation. After removal of the Fmoc, the resin was washed once with the same volume of solvent used for coupling (0.8 ml for method *b*; 1.2 mL for method *c*) 10 min under US irradiation. Upon the assembly of peptide sequences, the resin was thoroughly washed and dried. Hence, the peptides were detached from the resin by treating with TFA/TIS/H_2_O (95:2.5:2.5 v/v/v) on magnetic stirrer plate (200 rpm), recovered by precipitation with chilled Et_2_O (10 mL), and then centrifuged (6000 rpm × 15 min). The supernatants were carefully removed and the resulting white to pale yellow-coloured amorphous solids were dried and dissolved in Water/ACN (9:1) to be analysed by reverse phase HPLC**.**

**
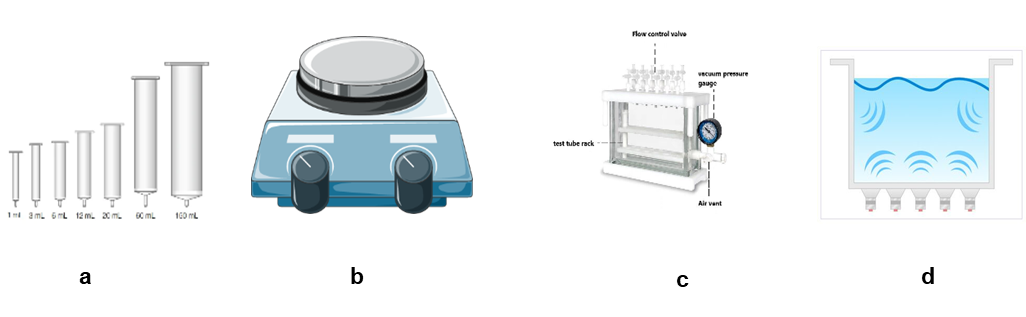
**

**Figure S1**. The laboratory equipment used for the US-SPPS: (**a**) polypropylene tube equipped with filter, top cap, and stopper or PTFE universal stopcock when needed; (**b**) magnetic stirrer plate; (**c**) vacuum manifold for solid-phase extraction (SPE); (**d**) ultrasonic bath equipped with timer control for 1-15 minutes and continuous (∞) operations and built-in heating control (30-80 °C thermostatically adjustable).

**2.1 Optimization and SUS-SPPS of peptide sequences 1-7.**

Peptide sequences were grown on: peptides **1** (methods *a*, *b*, *c*, *e*, Table S1) and **2** (methods *b* and *e*, Table 1) on Rink amide resin 0.64 mmol/g; 78 mg, 0.05 mmol; peptides **3-5** (methods *c* and *f*, Table 1) on Rink amide resin LL 0.29 mmol/g; 172 mg, 0.05 mmol; peptide **6** (method *b* and *e*, Table S1) on Wang resin 0.64 mmol/g pre-loaded with Fmoc-Leu amino acid; 39 mg, 0.025 mmol, peptide **7** (methods *b* and *e*, Table S1) on 2-CTC resin 0.50 mmol/g pre-loaded with Fmoc-Leu amino acid; 100 mg, 0.05 mmol.

**Table S1**

| **Method** | **Coupling** | **Capping** | **Deprotection** | **Washing** | **Ultrasound** |
| --- | --- | --- | --- | --- | --- |
| ***a*** | DMF 400 μL,  15 min | 2%, 3 min | 100 μL, 2 min | 400 μL,  5 min | YES |
| ***b*** | DMF 800 μL,  15 min | 2%, 3 min | 200 μL, 2 min | 800 μL,  10 min | YES |
| ***c*** | DMF 1200  μL, 15 min | 2%, 3 min | 300 μL, 2 min | 1200 μL  10 min | YES |
| ***d*** | DMF 400 μL,  15 min | 2%, 3 min | 100 μL, 2 min | 400 μL,  5 min | NO |
| ***e*** | DMF 800 μL,  15 min | 2%, 3 min | 200 μL, 2 min | 800 μL,  10 min | NO |
| ***f*** | DMF 1200 μL, 15 min | 2%, 3 min | 300 μL, 2 min | 1200 μL, 5 or 10 min | NO |
|  |  |  |  |  |  |

1. **Fmoc-KFRFD**; tR =10,24 [analytical HPLC/Gradient 2]; ESI-MS m/z calcd for molecular formula C_49_H_60_N_10_O_9_, m/z: 932.45, found: 932.65, 467.75 [M+2H/2]^+^;
2. **YNWNSFGLRF (Kisspeptin10)**; tR = 9.54 min [analytical HPLC/Gradient 1]; ESI-MS m/z calcd for molecular formula, C_63_H_84_N_17_O_14_ m/z: 1302.63, found: 1301,80, 652.00 [M+2H/2]^+^;
3. **ARLDVASEFRKKWNKWALSR (PAMP_1-20_)** tR = 15.03 min [analytical HPLC/Gradient 1]; ESI-MS m/z calcd for molecular formula C_112_H_179_N_36_O_27_ m/z: 2461.89, found 1229.60 [(M+2H)/2]^+^, 820.60 [(M+3H)/3]^+^, 616.15 [(M+4H)/4]^+^;
4. **Y-Aib-Aib-FL (Aib-Enkephaline)**; tR = 16.35 [analytical HPLC/Gradient 2]; ESI-MS m/z calcd for molecular formula C_32_H_46_N_6_O_6_ m/z: 610,349, found 611.25 [M+H]^+^, 633.20 [M+Na]^+^;
5. **VQAAIDYING (ACP _65-74_)**; tR = 13.61 [analytical HPLC/Gradient 2]; ESI-MS m/z calcd for molecular formula C_47_H_76_N_13_O_15_ m/z: 1062.55, found 1061.35, 532.10 [(M+2H)/2]^+^;
6. **DRVYIHPFHL-COOH (Angiotensin-I)**; Purity 92%; tR = 15.20 min [analytical HPLC/Gradient 1]; ESI-MS m/z calcd for molecular formula, C_62_H_90_N_17_O_14_ m/z: 1296.69, found 1294,90, 648.95 [(M+2H)/2]^+^;
7. **FRYKRL-COOH**; tR= 9.82 [analytical HPLC/Gradient 2]; ESI-MS m/z calcd for molecular formula C_42_H_67_N_13_O_8_ m/z 881,523, found 881,90, 442,25 [(M+2H)/2]^+^.
8. **HPLC Chromatograms and Mass Spectra**

**Figure S2.** Cromatogram of Fmoc-KFRFD (entry 1) obtained by RP-HPLC (Shimadzu Prominance UFLC, SPD-M20A/DGU20A3R/LC-20AD) on a Phenomenex Kinetex column (C18, 150 mm × 4.6 mm, 5 μm, 100 Å). Purity check/Gradient 1.

min

5,0

7,5

10,0

12,5

0

100000

200000

300000

400000

500000

uV

600000


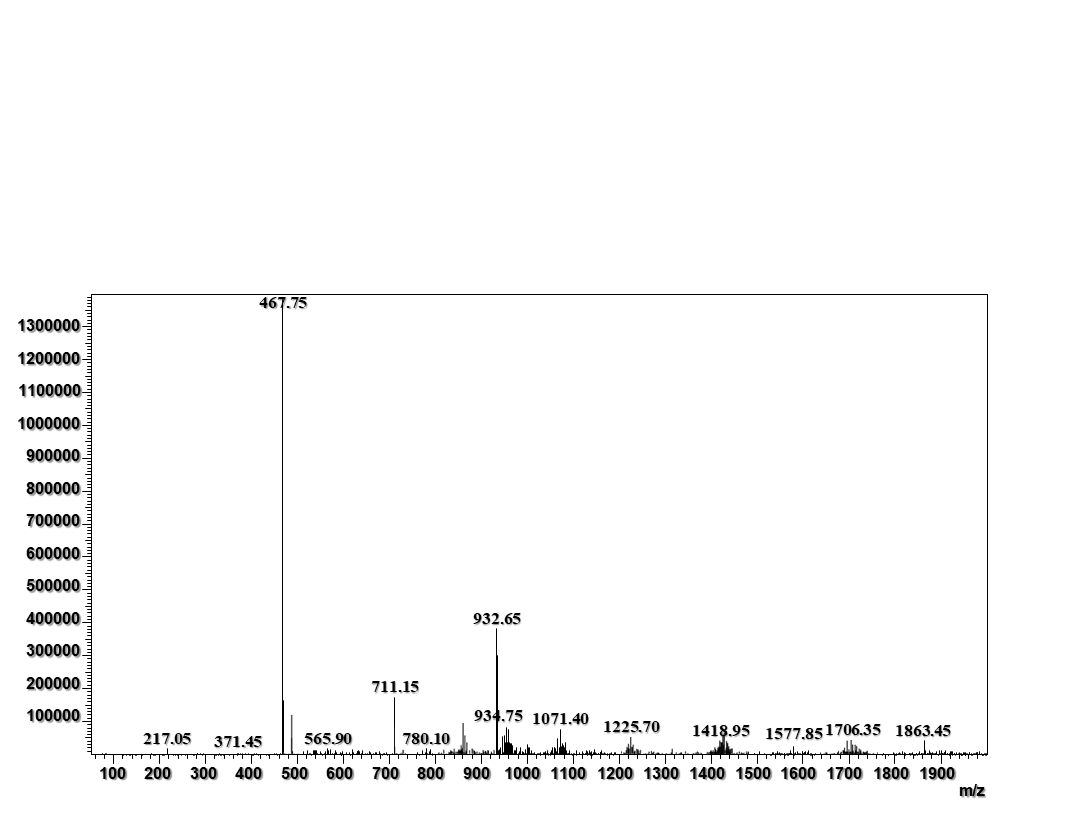


**Figure S3.** ESI-MS spectrum of peak at tR = 10.24 min resulted from representative chromatograms


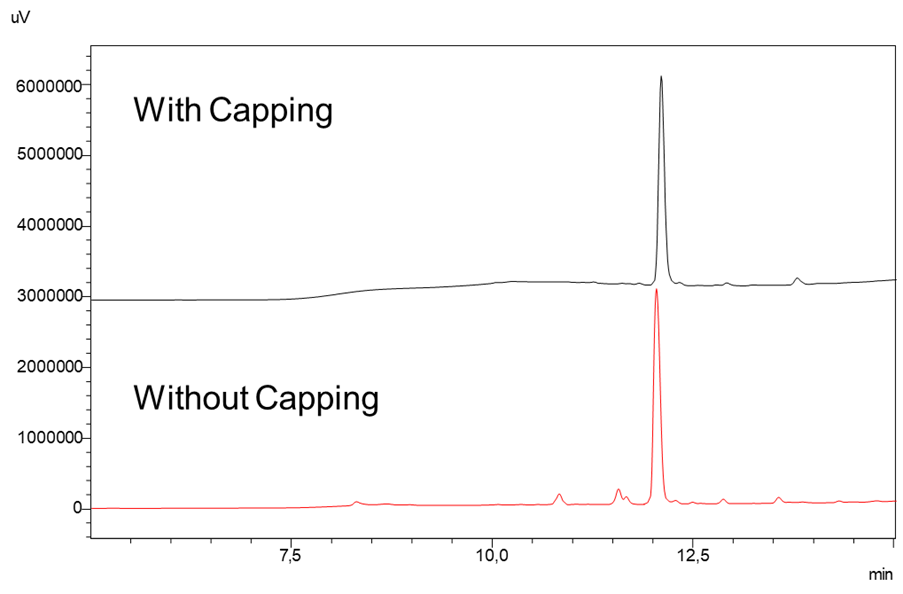

**Figure S4.** RP-HPLC Cromatograms of Fmoc-KFRFD (entry 1) obtained with and without capping step. (Shimadzu Shimadzu Model SPD-40V) on Phenomenex Kinetex column (C18, 150 mm × 4.6 mm, 5 μm, 100 Å). Purity check/Gradient 1.


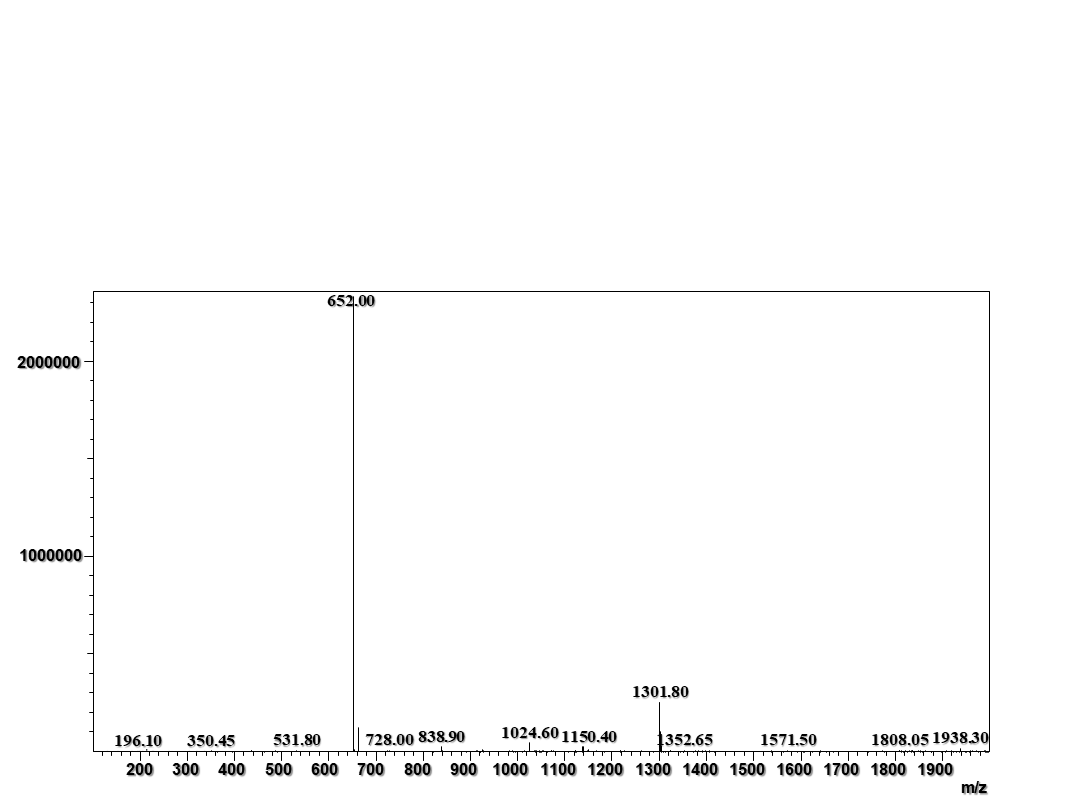
**Figure S5.** Chromatogram of Kisspeptin (entry 2) obtained by RP-HPLC (Shimadzu Model SPD-40V) on a Phenomenex Kinetex column (C18, 150 mm × 4.6 mm, 5 μm, 100 Å). Purity check/Gradient 1.

min

5,0

7,5

10,0

12,5

0

100000

200000

300000

400000

500000

uV

600000

**Figure S6.** ESI-MS spectrum of peak at tR = 9.54 min resulted from representative chromatograms


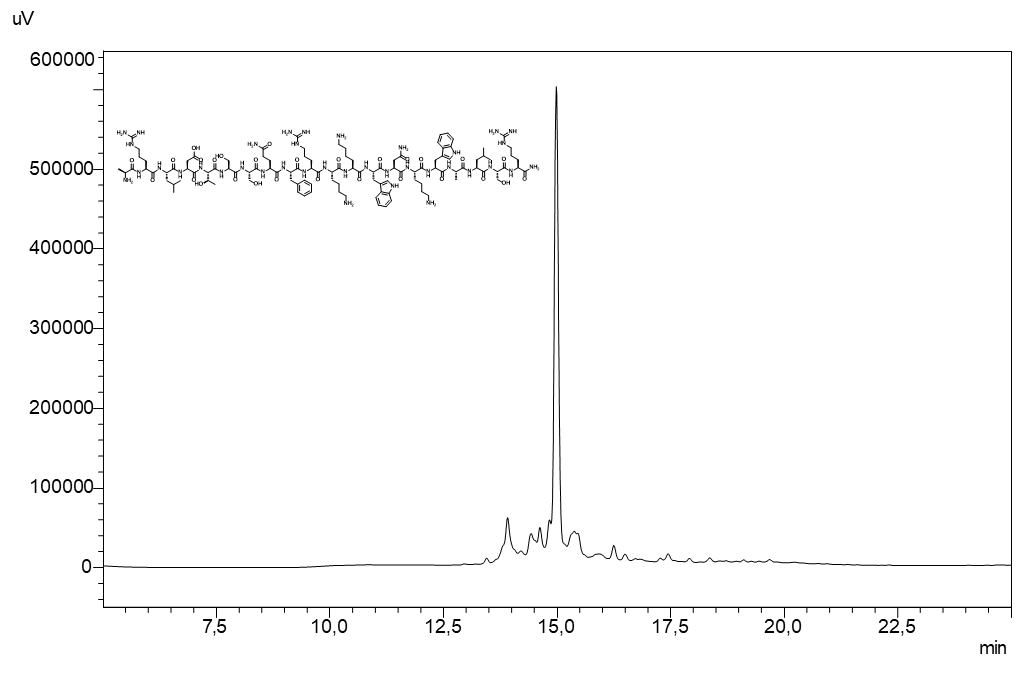


**Figure S7.** Chromatogram of PAMP_1-20_ (entry 3) obtained by RP-HPLC (Shimadzu Model SPD-40V) on a Phenomenex Kinetex column (C18, 150 mm × 4.6 mm, 5 μm, 100 Å). Purity check/Gradient 2.


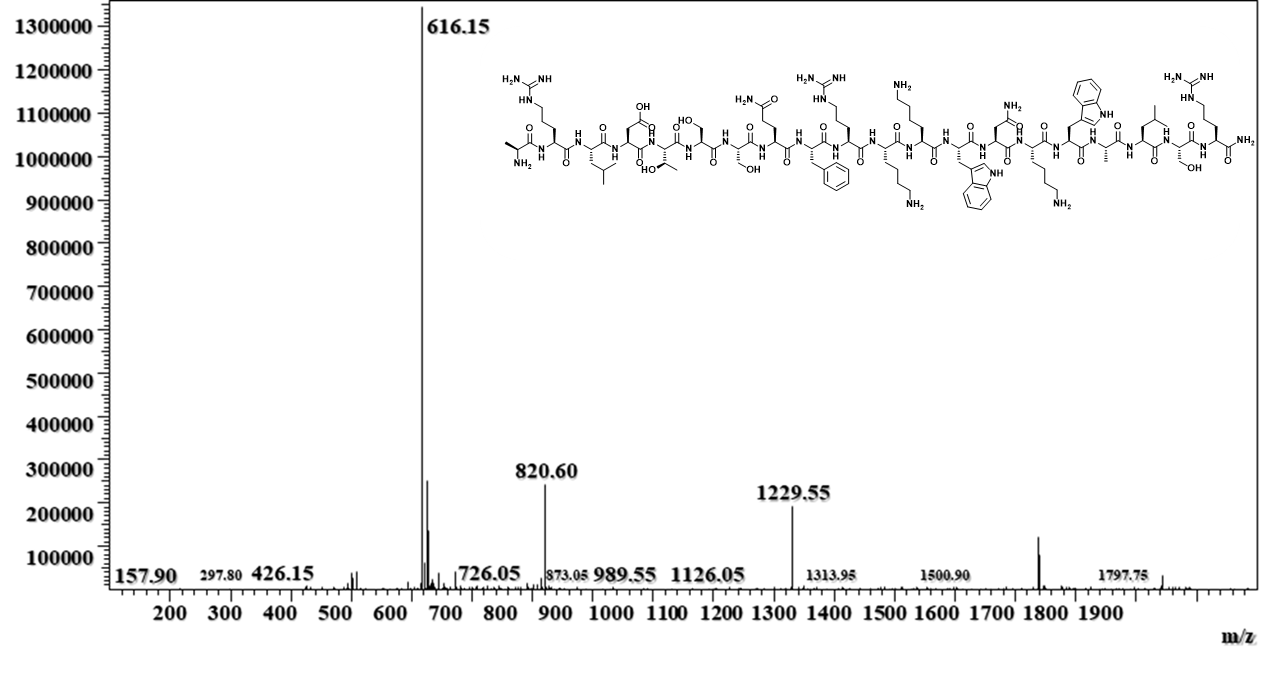


**Figure S8.** ESI-MS spectrum of peak at tR = 15.03 min resulted from representative chromatograms

**Figure S9.** Chromatogram of AIB-Enk (entry 4) obtained by RP-HPLC (Shimadzu Model SPD-40V) on a Phenomenex Kinetex column (C18, 150 mm × 4.6 mm, 5 μm, 100 Å). Purity check/Gradient 2.

min

7,5

10,0

12,5

15,0

17,5

20,0

22,5

0

100000

200000

300000

400000

500000

uV

600000


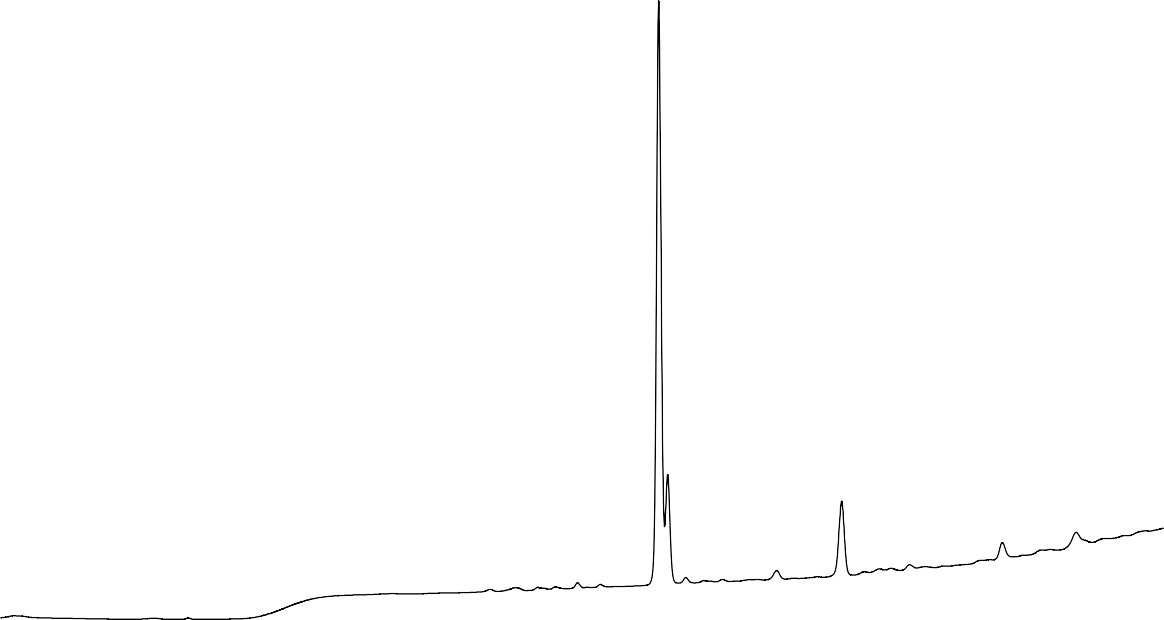


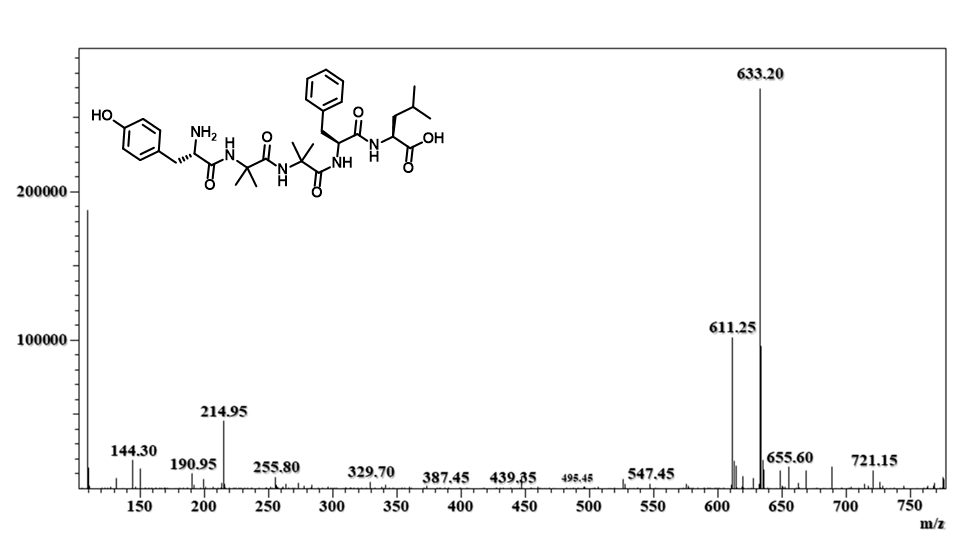


**Figure S10.** ESI-MS spectrum of peak at tR = 16.35 min resulted from representative chromatograms


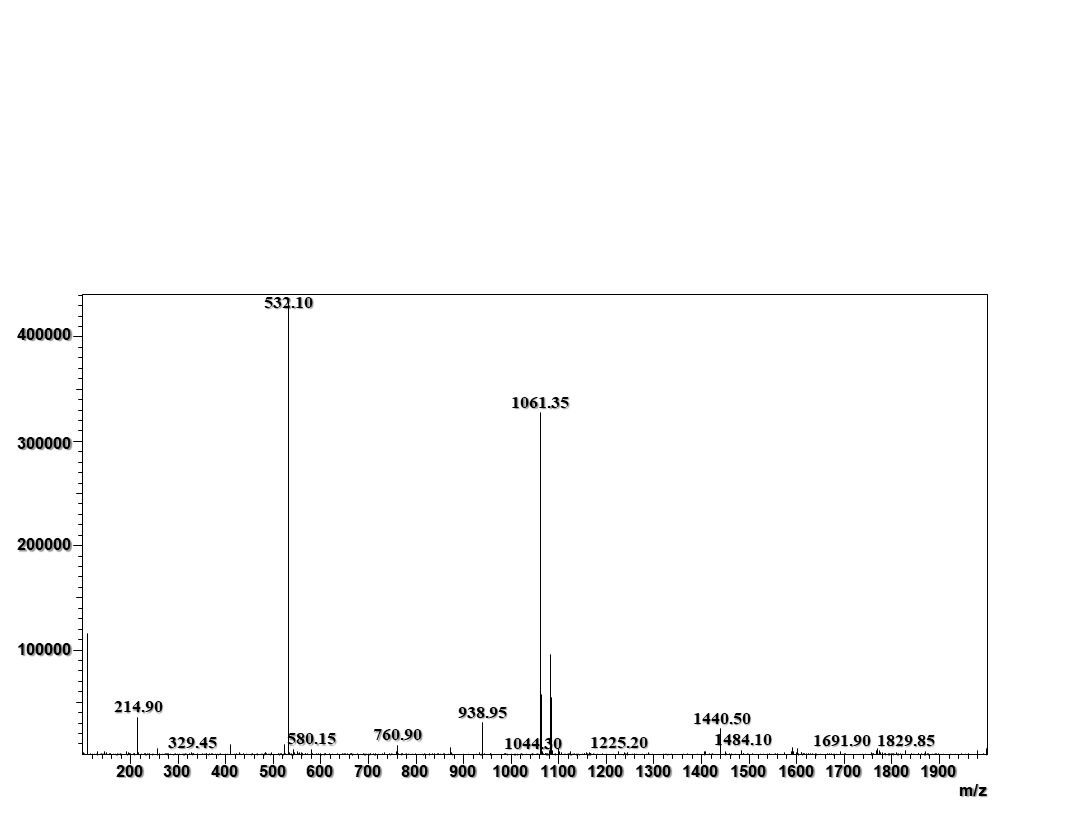

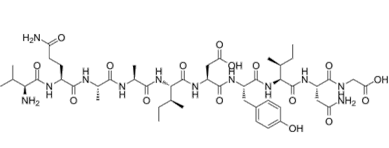
**
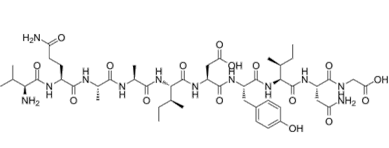
Figure S11.** Chromatogram of ACP _(65-74)_ (entry 5) obtained by RP-HPLC (Shimadzu Model SPD-40V) on a Phenomenex Kinetex column (C18, 150 mm × 4.6 mm, 5 μm, 100 Å). Purity check/Gradient 2.

min

7,5

10,0

12,5

15,0

17,5

20,0

22,5

0

10000

20000

30000

40000

50000

uV

60000

**Figure S12**. ESI-MS spectrum of peak at tR = 13.61 min resulted from representative chromatograms

**
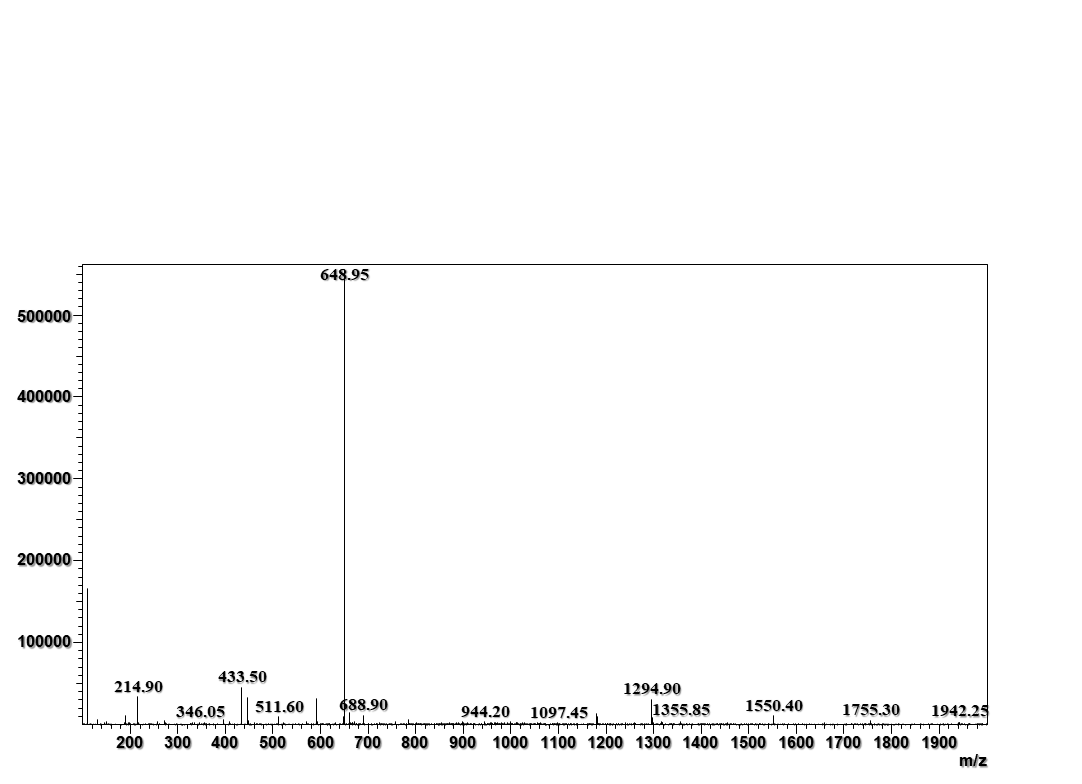
****Figure S13.** Chromatogram of Angiotensin I (entry 6) on Wang obtained by RP-HPLC (Shimadzu Model SPD-40V) on a Phenomenex Kinetex column (C18, 150 mm × 4.6 mm, 5 μm, 100 Å). Purity check/Gradient 2.

min

7,5

10,0

12,5

15,0

17,5

20,0

22,5

0

100000

200000

300000

400000

500000

uV

600000

**Figure S14.** ESI-MS spectrum of peak at tR = 15.20 min resulted from representative chromatograms

**Figure S15.** Chromatogram of Leu-Arg-Lys-Tyr-Arg-Phe (entry 7) on 2-CTC resin obtained by RP-HPLC (Shimadzu Model SPD-40V) on a Phenomenex Kinetex column (C18, 150 mm × 4.6 mm, 5 μm, 100 Å). Purity check/Gradient 1.

min

5,0

7,5

10,0

12,5

0

100000

200000

300000

400000

500000

uV

600000

**Figure S16.** ESI-MS spectrum of peak at tR = 9.82 min resulted from representative chromatograms


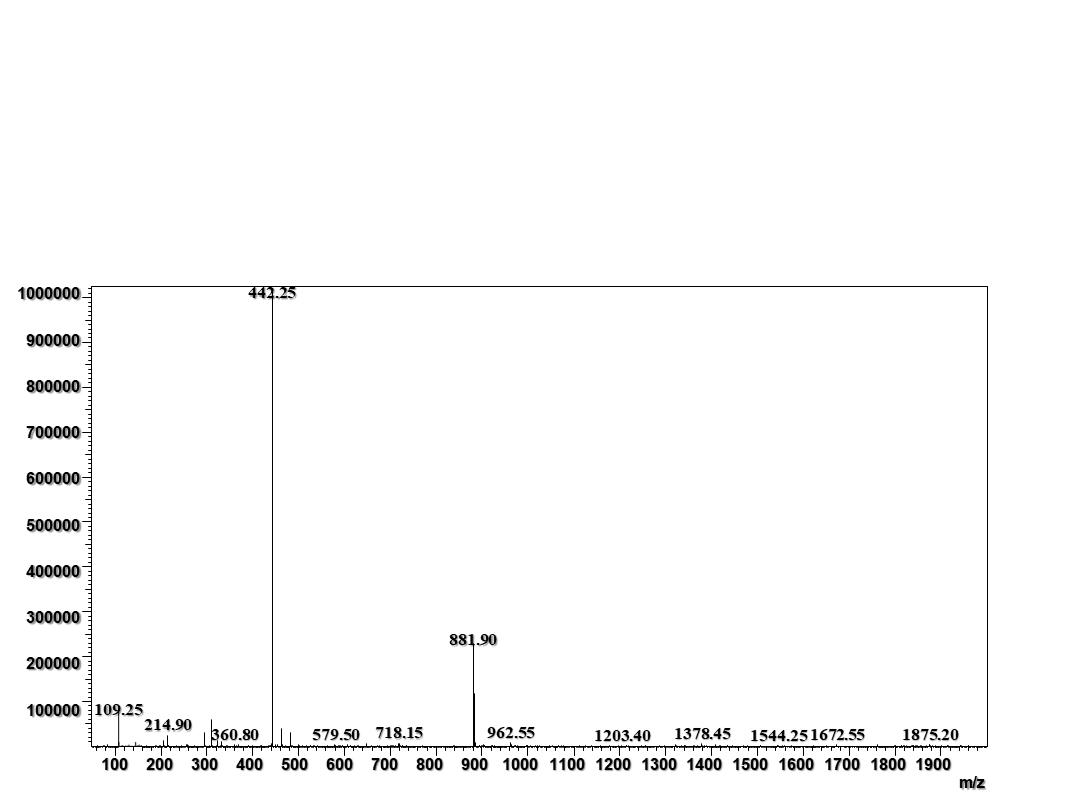

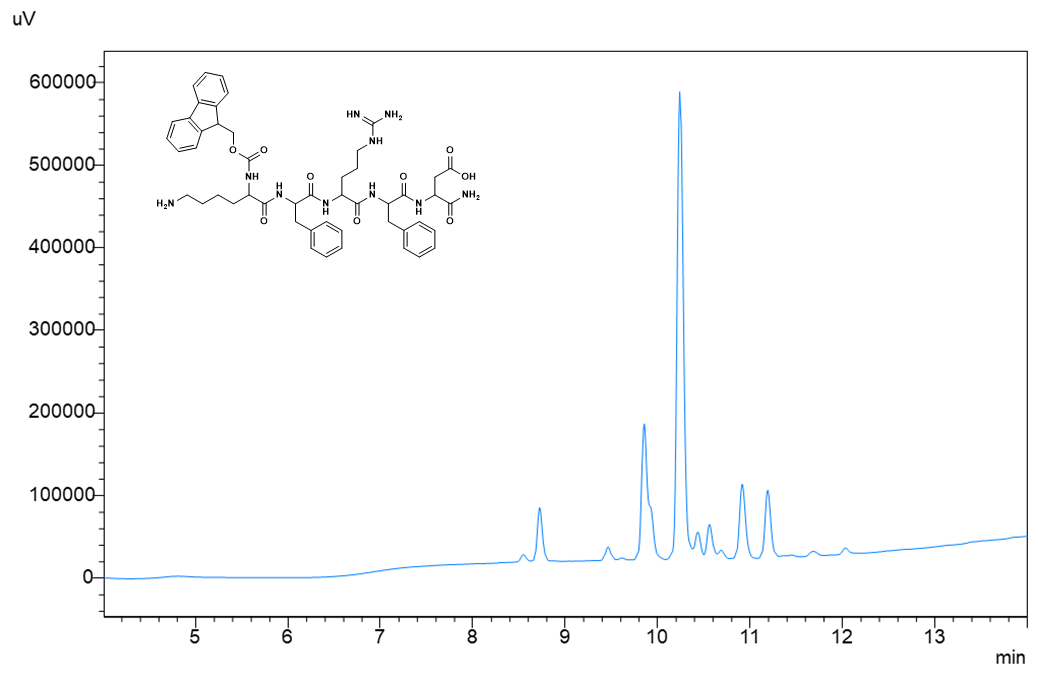


**Figure S17.** Chromatogram of Fmoc-KFRFD (method ***e***) obtained by RP-HPLC (Shimadzu Model SPD-40V) on a Phenomenex Kinetex column (C18, 150 mm × 4.6 mm, 5 μm, 100 Å). Purity check/Gradient 1.


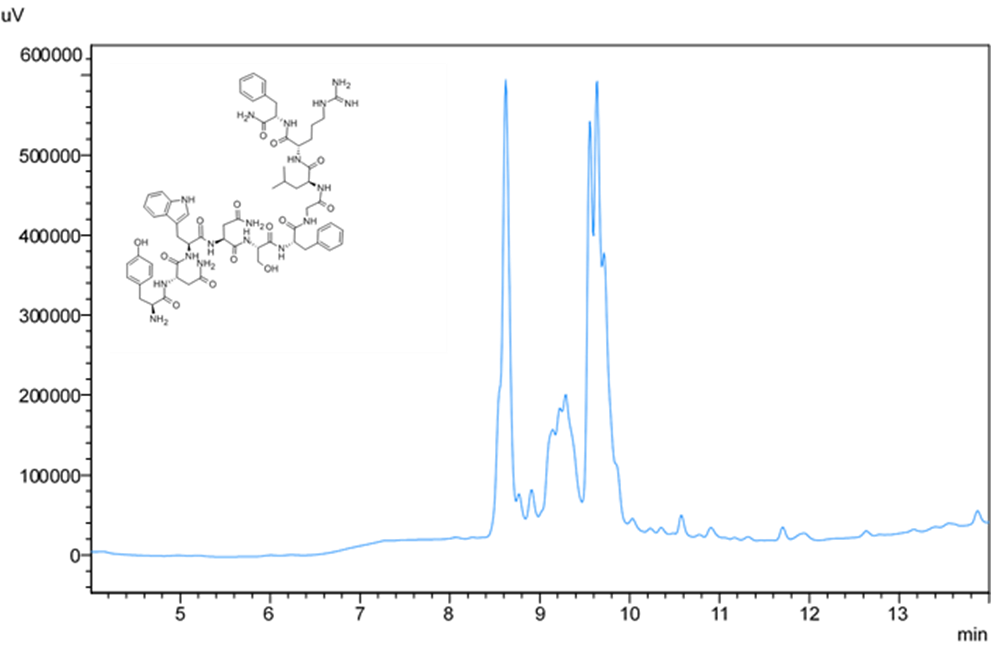


**Figure S18.** Chromatogram of Kisspeptin (method ***e***) RT obtained by RP-HPLC (Shimadzu Model SPD-40V) on a Phenomenex Kinetex column (C18, 150 mm × 4.6 mm, 5 μm, 100 Å). Purity check/Gradient 1.


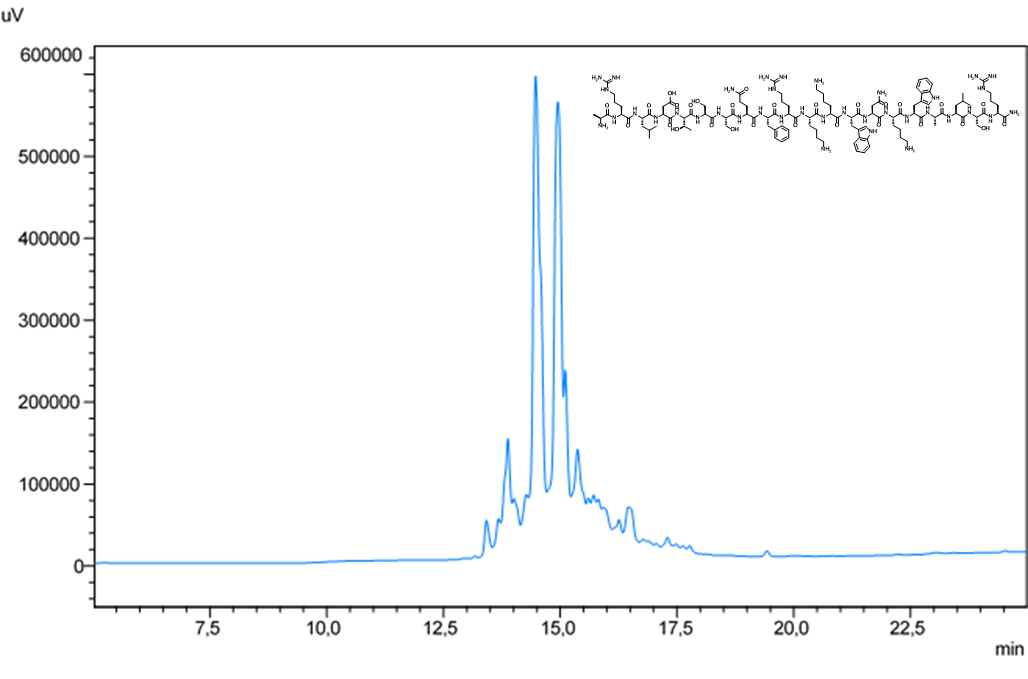


**Figure S19.** Chromatogram of PAMP1-20 (method f) obtained by RP-HPLC obtained by RP-HPLC (Shimadzu Model SPD-40V) on a Phenomenex Kinetex column (C18, 150 mm × 4.6 mm, 5 μm, 100 Å). Purity check/Gradient 2.


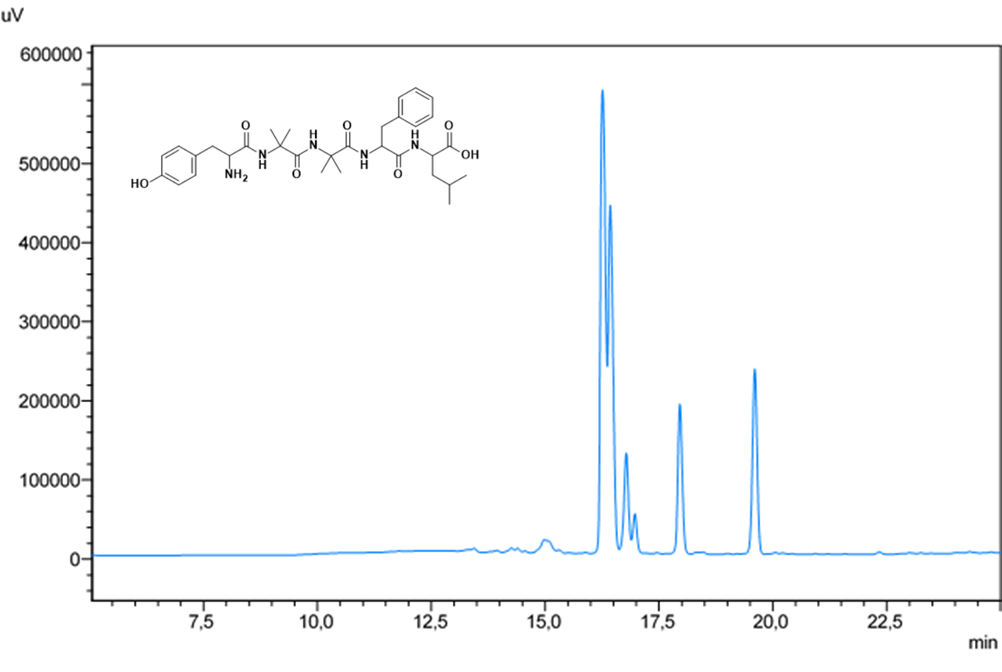
**Figure S20.** Chromatogram of Aib-Enk with (method ***f***) obtained by RP-HPLC obtained by RP-HPLC (Shimadzu Model SPD-40V) on a Phenomenex Kinetex column (C18, 150 mm × 4.6 mm, 5 μm, 100 Å). Purity check/Gradient 2.


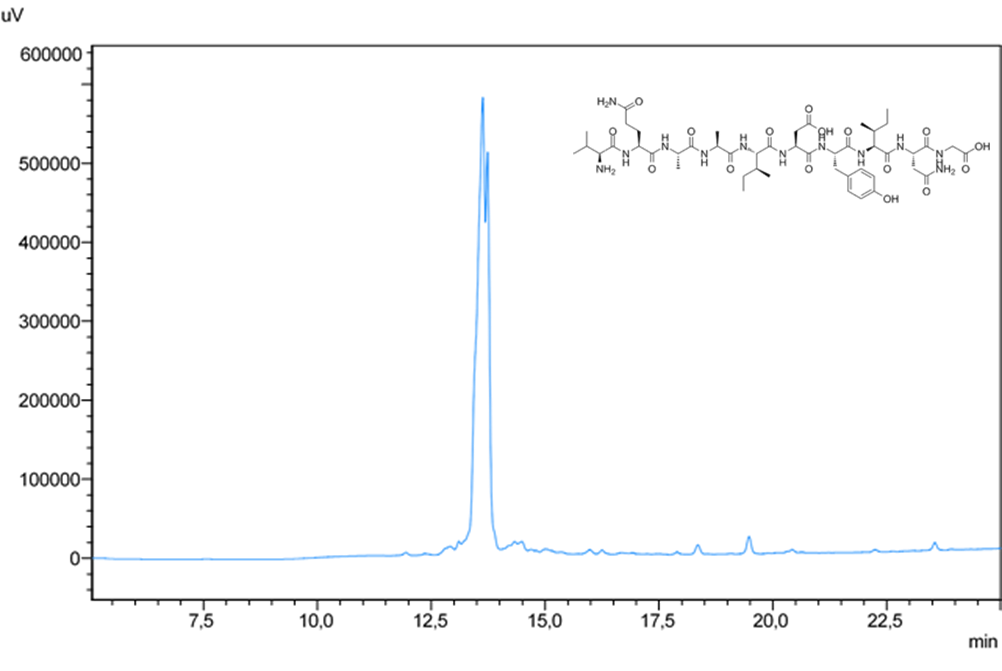
**Figure S21.** Chromatogram of ACP _(65-74)_ (method ***f***) obtained by RP-HPLC obtained by RP-HPLC (Shimadzu Model SPD-40V) on a Phenomenex Kinetex column (C18, 150 mm × 4.6 mm, 5 μm, 100 Å). Purity check/Gradient 2.


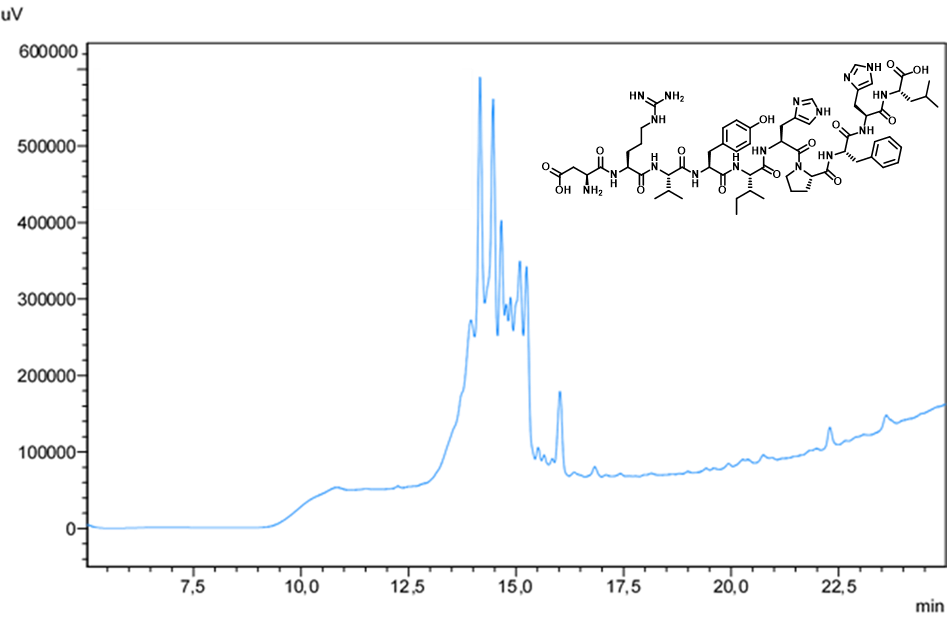
**Figure S22.** Chromatogram of Angiotensin I on Wang resin (method ***e***) obtained by RP-HPLC obtained by RP-HPLC (Shimadzu Model SPD-40V) on a Phenomenex Kinetex column (C18, 150 mm × 4.6 mm, 5 μm, 100 Å). Purity check/Gradient 2.


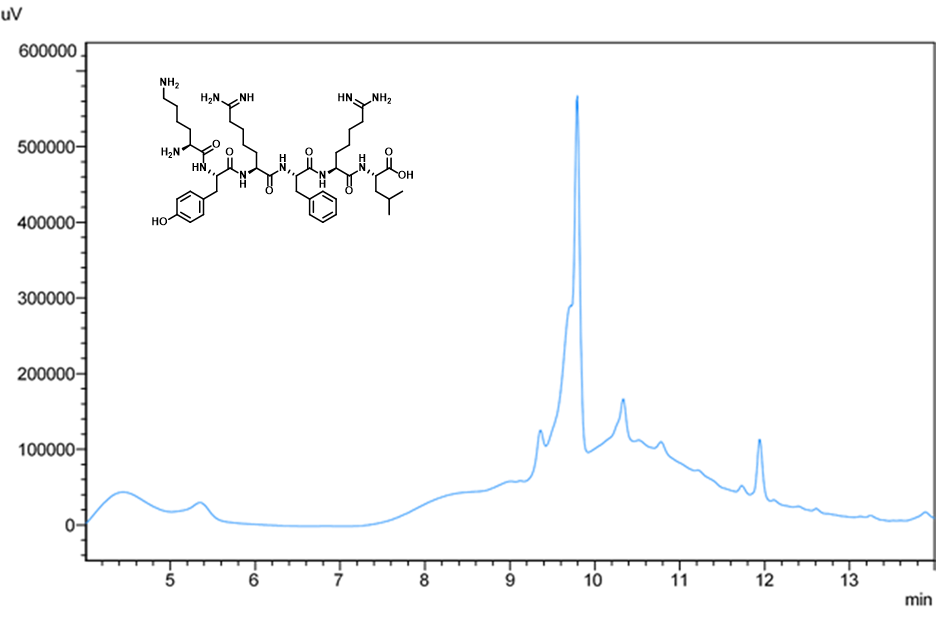


**Figure S23.** Chromatogram of 6-MER on 2-CTC resin (method ***e***) obtained by RP-HPLC (Shimadzu Model SPD-40V) on a Phenomenex Kinetex column (C18, 150 mm × 4.6 mm, 5 μm, 100 Å). Purity check/Gradient 1.
